# Machine Learning Advances in High-Entropy Alloys: A Mini-Review

**DOI:** 10.3390/e26121119

**Published:** 2024-12-20

**Authors:** Yibo Sun, Jun Ni

**Affiliations:** 1 State Key Laboratory of Low-Dimensional Quantum Physics, Department of Physics, Tsinghua University, Beijing 100084, China; syb21@mails.tsinghua.edu.cn; 2 Frontier Science Center for Quantum Information, Beijing 100084, China

**Keywords:** machine learning, deep learning, high-entropy alloys, multicomponent materials

## Abstract

The efficacy of machine learning has increased exponentially over the past decade. The utilization of machine learning to predict and design materials has become a pivotal tool for accelerating materials development. High-entropy alloys are particularly intriguing candidates for exemplifying the potency of machine learning due to their superior mechanical properties, vast compositional space, and intricate chemical interactions. This review examines the general process of developing machine learning models. The advances and new algorithms of machine learning in the field of high-entropy alloys are presented in each part of the process. These advances are based on both improvements in computer algorithms and physical representations that focus on the unique ordering properties of high-entropy alloys. We also show the results of generative models, data augmentation, and transfer learning in high-entropy alloys and conclude with a summary of the challenges still faced in machine learning high-entropy alloys today.

## 1. Introduction

The traditional alloying strategy is to add a small amount of a performance-enhancing element to a primary metal. However, the discovery of high-entropy alloys (HEAs) by Yeh and Cantor in 2004 introduced a different alloying strategy [1,2]. Since then, HEAs have been attracting widespread research interest not only due to their excellent mechanical properties but also due to their unique magnetic, electrical, and chemical properties compared to conventional alloys [3,4,5,6,7,8]. Because these materials have high configurational entropy, which is thought to be responsible for their stability, these alloys are named HEAs [1,9]. HEAs are also often referred to as multicomponent alloys, multi-principal-element alloys, compositionally complex alloys, or complex concentrated alloys [10] because the role of configurational entropy is not always as important as originally envisioned [9,11,12]. The novel alloy synthesis strategy of combining multiple primary elements indicates promising avenues for investigating the vast unexplored chemical compositional space beyond the corners of chemical compositional maps. On the inspiration of the HEA concept, Senkov et al. proposed refractory high-entropy alloys (RHEAs) which consist of a variety of primary refractory metal elements, including W, Mo, Ta, V, Nb, etc. [13]. As a branch of HEAs, RHEAs not only inherit the core properties of HEAs but also exhibit advantageous properties at high temperatures, including enhanced strength [14,15], corrosion resistance [16,17,18], and oxidation resistance [19,20], due to their refractory elemental composition with a high melting point. Therefore, RHEAs are recognized as structural materials with potential applications in the aerospace, nuclear reactor, automotive, and other industries. Another appealing aspect of HEAs is that the varying occupancy of lattice positions by multiple principal elements produces varying degrees of randomness and order, which are usually classified as short-range order (SRO) and long-range order (LRO) [21,22]. The degree of order and disorder in HEAs can be tuned to enhance their mechanical properties [23,24,25]. HEAs have become one of the most exciting research directions in materials science, and branches of HEAs with unprecedented properties continue to emerge [26,27,28].

While the incorporation of multiple-principal elements offers substantial prospects for alloy design, it concurrently presents considerable challenges for theoretical modeling and simulation. First, experimental methods usually require multipurpose equipment and a lot of human power. The experimental process takes a long time for even a small fraction of HEAs. It is almost impossible to discover HEAs by trial-and-error experiments alone due to the extremely large compositional space. Even if substitute models such as phase formation rules [29] and ductility criteria [30] are relied upon to guide effective exploration of the vast compositional space, these empirical rules are challenging for HEAs due to the large variety of chemicals involved. Second, the construction of empirical atomic interaction models is hindered by the considerable number of chemical interactions involved. It can be reasonably considered that the number of interactions will increase exponentially with the introduction of higher-order interactions [22]. This will render the traditional cluster unfolding methods [31] and the embedded atom method [32] susceptible to overfitting. Finally, although computational methods such as Density Functional Theory (DFT) [33], molecular dynamics (MD) simulation [34], and phase diagram calculation (such as CALPHAD) [35] have been successfully used to explore HEAs, the high computational cost, time consumption, and uncertainty of these methods have severely hindered their application to HEAs.

Over the past decade, machine learning has seen rapid growth in its performance as an artificial intelligence (AI) tool [36]. This development has a profound impact on numerous fields within computer science, including computer vision and natural language processing [37]. Furthermore, machine learning provides novel possibilities for the development of non-computer scientific fields, including protein structure prediction [38], medical image analysis [39], particle signal detection [40], and universe analysis [41]. Machine learning also has significant advancements in material science. The calculations of interatomic potentials using ML models are usually much faster than DFT, and the results are close to the interatomic potentials calculated by DFT [42,43,44]. These application break through the original limitations of computational speed and computer memory for DFT, allowing researchers to simulate materials with more than millions of atoms with near-DFT accuracy [45,46]. This ability is sufficient to simulate the properties of HEAs at the nanoscale and is particularly important for exploring the complex long-range structure, which impacts the excellent mechanical properties in HEAs [47,48]. In addition to simulations, machine learning methods provide viable solutions for predicting material properties and screening potential candidate materials [49]. The intricate, non-linear interconnections between structure and property are challenging to convey through mere human observation. The efficacy of machine learning is contingent upon its capacity to elucidate intricate patterns. With the help of machine learning, researchers can explore the huge range of possible materials more quickly. Machine learning has emerged as a promising tool for addressing the challenges inherent to the theoretical modeling of HEAs [50] and has been successful in physical property prediction [51,52], atomistic simulations [53,54,55], phase classification [56], and material design [57,58,59].

We focus on the application of machine learning to HEAs. This review begins by outlining the process of data collection, the selection of appropriate descriptors, the development of algorithms, and the subsequent analysis of performance in machine learning. This is presented in the order in which machine learning models are developed. Furthermore, each section provides a detailed account of the distinctive advancements in machine learning of HEAs, such as descriptors or algorithmic structures applicable to HEAs. Some of the advances are inspired by the high entropy or long-range structural randomness of HEAs, which react to properties that are unique to HEAs. In addition, we present some other special machine learning methods, including generative models, data augmentation, and transfer learning, and discuss their applications in the HEAs. The aforementioned algorithms are of significant assistance in enhancing the performance of machine learning. The relative strengths and weaknesses of different machine learning methods are compared. The final section discusses the challenges associated with machine learning methods in HEAs and, based on these challenges, offers insights into the future direction of machine learning.

## 2. General Model Process

Although machine learning is frequently a versatile and powerful tool, a single machine learning model is only applicable to a specific problem. This section describes the general process for developing a machine learning model, with a particular focus on recent advancements in the field of HEAs.

### 2.1. Data Collection

The first step in developing a machine learning model is to collect datasets. The dataset should comprise the targets of the problem and target-related, readily accessible inputs for utilization upon completion of the model development process. DFT represents a commonly employed method for the collection of datasets, offering high levels of generalizability. The dataset calculated by DFT can be employed not only to fit interatomic potentials in order to assist MD simulations but also to directly fit the energy, magnetic moment, and other properties of HEAs. Tran et al. calculate the formation energies and magnetic moments of hundreds of alloys in the FeCoNiCrMn/Pd system [60], delivering the dataset that can be used for the development of machine learning models. It is important that DFT datasets used for the same model use the same computational methods with the same parameters, in particular the pseudopotentials and energy cutoffs. This is to ensure that the energy differences between the data can realistically reflect the effects of atomic arrangements and make it easier for the model to learn the relationships. The size and distribution of the dataset is important for the model performance because machine learning models are always stronger at interpolation and weaker at extrapolation. In order to accelerate the DFT calculations of the dataset, the effective pair interaction (EPI) algorithm is developed by Liu et al. [61]. It is an Ising-like model with only effective pair interactions without considering high-order interactions [53]. The EPI algorithm can significantly accelerate the calculations of the DFT. Furthermore, the machine learning model based on the dataset of the EPI algorithm is an effective method for accelerating the MD simulation of HEAs [54]. For the prediction of material properties, it is also possible to download datasets directly from the computational database, including the Open Quantum Mechanics Database of Northwestern University [62], the Materials Project of Lawrence Berkeley National Laboratory [63], AFLOW [64], Materials Cloud [65], and so on.

Experimentation is also an important method of acquiring a dataset. Similar to DFT calculations, the dataset should be maintained under the same experimental conditions. There are some experimental datasets, such as the Pauling File [66], the High Throughput Experimental Materials [67], and the Materials Experiment and Analysis Database [68]. Some high-performance machine learning models of high-entropy solid solutions or alloys are built on these databases [51,69]. There are also some databases focusing on high-entropy alloy materials. For example, a database containing the mechanical properties and phases of 1545 high-entropy alloys is developed by Borg et al. [70,71,72] A database containing 1252 solid solutions and intermetallic compounds is also developed by Gao et al. [73] The dataset of Kube et al. contains 2425 quinary alloys [74]. A machine learning model for high-entropy alloy phase classification is constructed by Feng et al. [75] using the databases from Gao et al. [73] and Kube et al. [74] Feng then evaluated the performance of the model on the two databases, which again validates the importance of a sufficient amount of data for machine learning performance. Even with the use of transfer learning, a technique that improves performance with small datasets, the classification accuracy of the database with 355 data [73] is still lower than that of the database with 2425 data [74].

### 2.2. Descriptor Selection

Descriptors are the inputs to machine learning models. The essence of machine learning is to fit the relationship between descriptors and predictions. It is important to find descriptions that have strong correlations with predictions. In order to facilitate material development, elemental types and ratios are often considered. In machine learning, elemental types are often represented using one-hot encoding [76] or element embedding [77]. For the prediction of specific material properties, it is necessary to identify descriptors associated with specific material properties. In order to predict the hardness of HEAs, for example, the melting temperature of the alloy would be an appropriate descriptor, given that it is an indirect representation of the metallic bond strength [52]. Some empirical descriptors are often used, such as mixing enthalpy or mixing entropy [78]. A well-established method of descriptor selection is to filter from the vast descriptor space. Zhang et al. use a genetic algorithm to screen from 9 models and 70 descriptors [79]. Eventually, a Support Vector Machine with four descriptors, the average atomic number, the difference in electronegativities, covalent radii, and the boiling temperature, became the best combination of model descriptors for predicting the crystal structure of HEAs with an accuracy of more than 90% [79].

**Figure 1:**
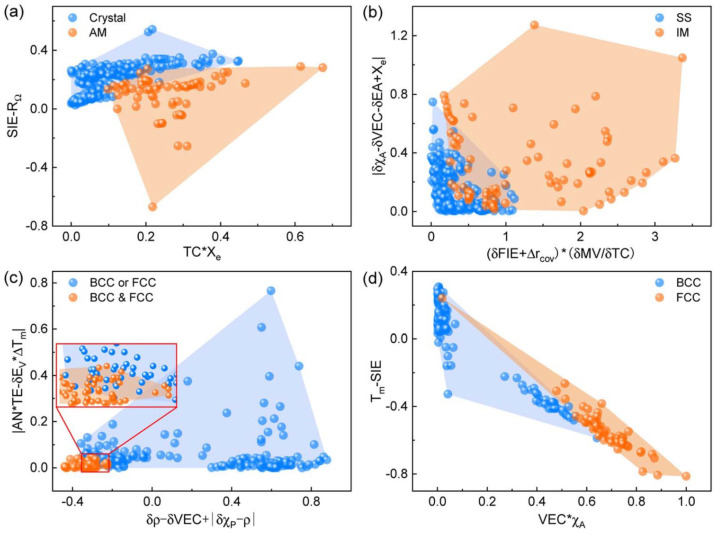
HEAs phase classification using SISSO descriptors as coordinates in Ref. [81]: (**a**) Classification of crystal and amorphous (AM). (**b**) Classification of intermetallic (IM) and solid solution (SS). (**c**) Classification of single-phase (BCC or FCC) and multi-phase (BCC and FCC). (**d**) Classification of BCC and FCC.

The sure independence screening and sparsifying operator (SISSO) is a data-driven approach that combines symbolic regression and compressed sensing to construct descriptors of target attributes. It is able to find the optimal descriptor through operations between low-dimensional descriptors [80]. Shang et al. complete the phase classification of HEAs using SISSO relying on only two descriptors [81]. And the descriptors have some physical significance, making it possible to draw accurate, physically interpretable 2D phase diagrams in Figure 1 [81].

Based on the SRO in HEAs, liu et al. propose a descriptor based on Voronoi analysis and Shannon entropy (VASE) [82]. The Voronoi analysis is employed as a basis for introducing Shannon entropy, which is used to directly represent the information about the spatial arrangement of atoms. VASE is capable of responding effectively to the disordered atomic occupancies in HEAs. The coefficient of determination ($R^{2}$) for the prediction of the formation energy of the FeCoNiAlTiCu system is 0.918. It is an efficient representation based on the unique arrangement of atoms in HEAs. It is worth mentioning that the model chosen by liu et al. for VASE is a Light Gradient Boosting Machine [83], which shows that with strongly correlated descriptors, even relatively simple models have the potential to achieve good performance.

### 2.3. Model Selection and Development

The efficacy of machine learning algorithms varies according to their specific characteristics and inherent limitations. It is essential to select an appropriate algorithm in accordance with the specific task objectives and the size of datasets. Machine learning algorithms can be divided into two main groups. One group comprises simple regression models and ensemble learning models. Such models include support vector machine (SVM) [84], classification and regression tree (CART) [85], k-nearest neighbor (KNN) [86], etc. These ensemble learning algorithms are relatively simple, easier to understand, and easier for researchers to find patterns within the model. The other group is deep learning algorithms, represented by artificial neural networks (ANNs) [37]. An ANN consists of multiple functional layers, and each layer consists of multiple neurons. The simplest ANN is the multi-layer perceptron, which is a feed-forward network with input, output, and hidden layers comprising fully connected networks. Other frequently utilized neural networks include convolutional neural networks (CNNs) [87], recurrent neural networks (RNNs) [88], and Transformer [89], which is employed in the context of large language models. In contrast to ensemble learning, deep learning models, while they can exhibit high model performance, are typically too complex to be readily comprehended. Cheng et al. use multiple machine learning algorithms to predict the hardness of HEAs [90]. The root mean square errors (RMSEs) on the test set of each model are shown in Table 1. In their work, ANN performs better than SVM and KNN, which shows the excellent performance of deep learning. Nevertheless, it has been demonstrated that simple machine learning may perform better compared to deep neural network models when the dataset size is limited [91]. This fact can be attributed to the propensity of complex networks to exhibit overfitting when confronted with relatively limited datasets.

**Figure 2:**
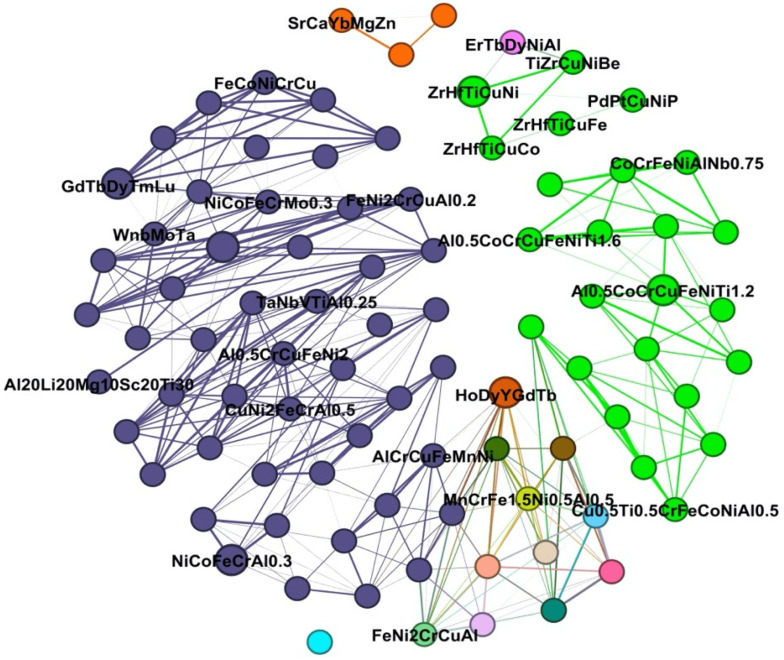
Correlation and similarity between HEA materials obtained using HEA interaction network in Ref. [96].

Graph neural networks (GNNs) have been extensively developed and have demonstrated remarkable performance in a multitude of tasks within the field of materials science [92]. Graphical representations are highly versatile in capturing the local chemical environment, which is the reason for the success of GNNs in materials science. The graphical representations defined by GNNs are directly encoded by the representation of atoms as nodes and chemical bonds as edges. Additionally, a GNN possesses the capacity to incorporate physical information such as charge and spin. There are many successful GNN-based machine learning models for materials science, such as Schnet [93], CGCNN [94], and MEGNet [95]. The capacity of GNN to represent crystals enables these models to accurately characterize the material properties of a diverse array of systems, thereby attaining advanced performance. Ghouchan et al. propose a graph-based KNN method for predicting phases in HEAs [96]. In their work, Ghouchan proposes a definition of an HEA interaction network, in which each HEA material is represented as a node on the graph. The edges of the graph represent the correlation between materials, with a stronger correlation indicating a higher degree of similarity. This correlation is obtained through the calculation of the similarity between material descriptors. Ghouchan then uses KNN to predict the phases of the HEAs. KNN first selects all the neighbors of each compound in the network and predicts the phase of the target compound using correlation as the voting weight. A part of the HEA interaction network can be seen in Figure 2. Each node represents a distinct material. Nodes of the same color are deemed to be highly similar. Materials of the same color usually belong to the same phase. This model demonstrates well the differences and similarities between HEA materials [96].

**Figure 3:**
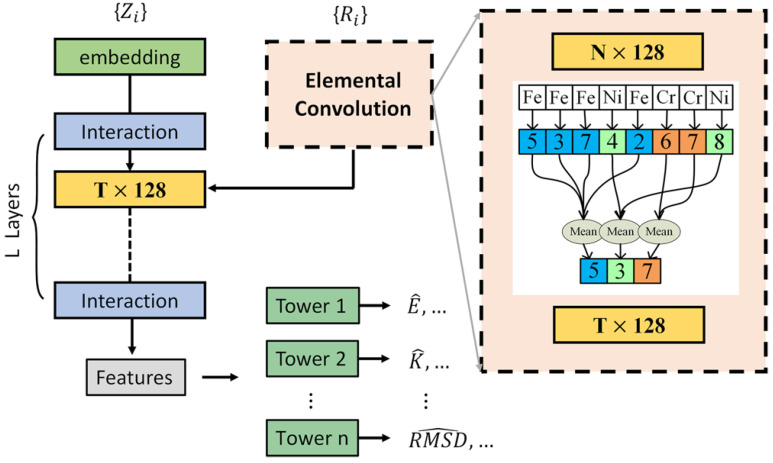
The framework of the ECNet model in Ref. [97]: The embedding layer serves the function of encoding the initial inputs derived from the atomic numbers. In the interaction block, a series of neural networks is employed for the purpose of transforming the crystal structures into atomic attributes. The elemental convolution operation entails the computation of the mean value of the atom-wise features, classified according to the atomic element type.

**Figure 4:**
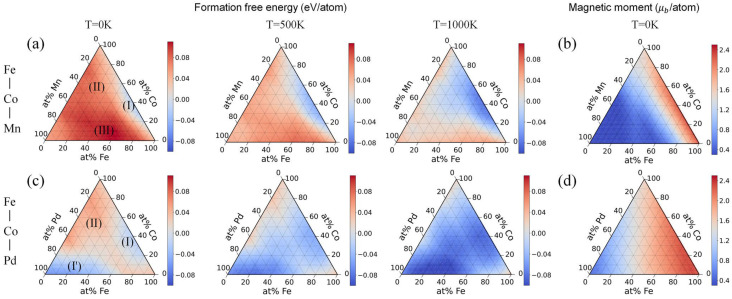
Ternary diagrams predicted by the ECNet in Ref. [97]: (**a**) formation free energies in FeCoMn system; (**b**) magnetic moments in FeCoMn system; (**c**) formation free energies in FeCoPd system; (**d**) magnetic moments in FeCoPd system. Areas I, II, III represent the stability of alloys from high to low.

Due to the absence of LRO, the properties of HEAs are influenced not only by the local environment of the atoms but also by long-range disorder. At the same time, when predicting the properties of a material, not all target properties are greatly influenced by the local structure of the atoms. Therefore, a machine learning model that can respond to long-range properties is important for HEAs. Wang et al. develop the elemental convolution graph neural network (ECNet) [97], which enables the element-wise features to function as both intermediate and final describers, thereby facilitating the extraction of knowledge pertaining to both atomic information and crystal structures. Furthermore, these features can be updated through the process of learning target material properties. The process of ECnet is referred to in Figure 3, and the atomic local information obtained from the graph network is converted into global elemental properties by elemental convolution to obtain long-range average properties. Wang predicts the formation free energies and magnetic moments of HEAs using ECNet and plots ternary diagrams in Figure 4. These findings contribute to the advancement of physical understanding of HEAs. This reflects another role of machine learning: resolving the difficulty of a near-infinite compositional space in material screening, especially in high-dimensional materials such as HEAs.

The effect of LRO on high-entropy alloys is considered in the work of Zhang et al. [98] The long-range structure of HEAs is a disordered combination of different local environments. Zhang et al. develop an aggregation module based on the GNN model to reflect long-range structures. The GNN learns representations of the local environments, which gains the SRO of HEAs, and the aggregation module randomly combines these representations into global representations of HEAs. The mean absolute errors in the prediction of the bulk modulus and Young’s modulus of HEAs after using the aggregation module are below 8 GPa and 11 GPa [98].

The interatomic potential is a function that describes the dependence of the potential energy on the atomic positions, which is a crucial aspect for molecular dynamics simulations. Recently, machine learning potentials have become a favorable tool for molecular dynamics simulations of complex materials [99]. In comparison to the traditional potentials, machine learning potentials exhibit two principal characteristics. Firstly, machine learning potentials adopt a data-driven methodology comprising training, validation, and testing phases, utilising datasets derived from first-principles calculations. It is beneficial for improving the accuracy of machine learning potentials. Secondly, machine learning potentials assume a malleable, rather than a fixed, functional form, thereby facilitating a systematic enhancement in accuracy [4]. Consequently, high-quality machine learning potentials are capable of describing the interatomic potentials of complex systems with a minimal number of parameters and achieving a level of accuracy that is comparable to that of quantum mechanical methods such as DFT [100]. There are already many proven machine learning potentials, such as the Gaussian approximation potential [101], the spectral neighbor analysis potential [102], Behler–Parrinello neural-network potential [103], deep potential [104], atomic cluster expansion [105], the moment tensor potential [106], and the neuroevolution potential [107,108]. In the Gaussian approximation potential, the total energy is predicted by the Gaussian process regression method, which measures the degree of similarity to the reference atomic environment. Different kernel functions can be chosen depending on the different reference atomic environments. Constructing machine learning potentials that satisfy symmetry to improve machine learning prediction accuracy is also commonly used in HEAs [109], such as the EPI mentioned in Section 2.1 [53,54], low rank potential [110,111], Gaussian approximation potential [112], and moment tensor potential [113]. Li et al. use the spectral neighbor analysis potential to study the Peierls stress for both screw and edge dislocation in the equiatomic NbMoTaW HEAs [48]. They find strong evidence of Nb segregation to the grain boundaries of the NbMoTaW MPEA by machine learning potential-assisted MD simulation. These machine learning potentials can also be used as descriptors to predict properties. Pandey et al. predict the hardness and modulus of MoNbTaTiW alloys using moment tensor potential-based machine learning [114]. Song et al. develop a neuroevolution potential model with 16 elemental metals and their alloys and achieve the transfer from unary and binary materials to multi-component alloys [115]. Their model predicts the formation energy of multi-component alloys and studies the plasticity and primary radiation damage on MoTaVW alloys.

Another approach to constructing machine learning potentials is to directly predict interatomic potentials from symmetric atomic structure inputs. The GNN represents the atomic structure as an undirected graph that directly satisfies the translational invariance and rotational covariance, which makes GNN a powerful tool for predicting machine learning potentials. The characteristics of GNN that use crystal structure to predict material properties are applicable to assist the MD simulation. Cheol et al. use GNN to predict interatomic forces directly from material structures. The predicted interatomic forces are used in MD simulations to obtain new structures [42]. Wu et al. use GNN to predict the interatomic potential to obtain MD simulation lattices with less than 1% error [116]. This process eliminates the need to artificially design material features to satisfy translationally invariant and rotationally covariant and speeds up calculations.

Machine learning potentials can be used to quantify and understand SRO in complex materials [117]. There are millions of possible SRO configurations in a quinary alloy, and it is difficult to traverse these configurations using DFT. Therefore, machine learning potential-assisted MD simulation becomes a powerful tool to study SRO in complex materials. Chen et al. use the neuroevolution potential to study SRO in GeSn alloys [118]. With machine learning potentials enhancing the scale of the study, they find the coexistence of two types of SROs in GeSn alloys.

### 2.4. Performance Analysis

In order to ensure an objective and accurate evaluation, it is essential that the test samples are representative and uncorrelated with the training samples. Otherwise, this may result in artificially high scores. In the context of classification tasks, various metrics are commonly employed to assess model performance, including recall, precision, the area under the curve, and receiver operating characteristic curves. In the case of regression tasks, RMSE and $R^{2}$ score are frequently utilized [119].

Although well-trained ML models are capable of making accurate predictions, in many instances, the interpretability of their model performance is also a crucial consideration. The interpretable predictions provided by machine learning models can offer insights into physics. Pei et al. analyze the contribution of various descriptors to the ductile Mg alloys by machine learning and show that the two mechanisms, dislocation nucleation and dislocation cross-slip, respectively, are always strongly correlated, rather than only one being dominant, as is often assumed [69]. Pei et al. also propose a solid solution formation rule based on machine learning that contains important features such as bulk modulus [51], which is not considered in the previous Hume-Rothery rules [120]. SHapley Additive exPlanations (SHAP) is also an effective method for machine learning interpretation [121]. SHAP can provide physical understanding for machine learning by giving the importance and contribution of each descriptor.

**Figure 5:**
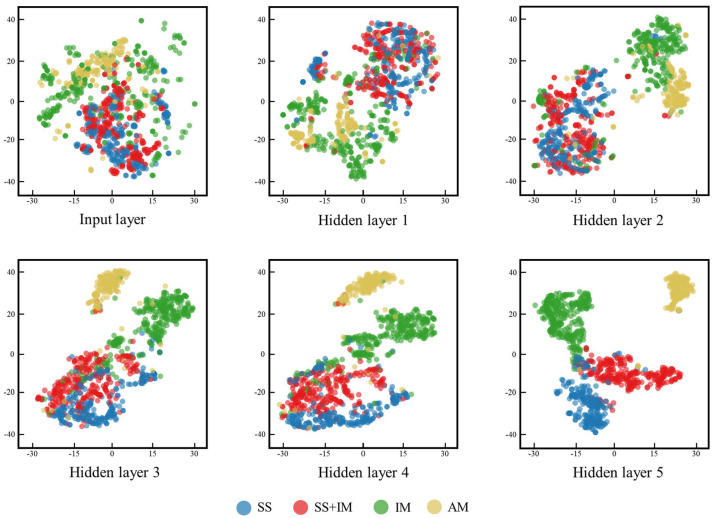
Feature visualization using the t-SNE algorithm in Ref. [123]. The model in Ref. [123] contains a total of five hidden layers. The original inputs to the model and the output results of the five hidden layers are visualized as two-dimensional distributions using the t-SNE algorithm, respectively. The color of the points represents the phase of the alloys: blue, solid solution; red, mixed phase of solid solution and intermetallic; green, intermetallic; yellow, amorphous.

The interpretability of deep neural networks is often hindered by their non-linearity and high dimensionality. At present, one of the most established methods for visualizing high-dimensional data is the t-distributed stochastic neighbor embedding (t-SNE) algorithm [122]. The t-SNE has been successfully applied in the visualization of phase classification in HEAs [55]. The t-SNE algorithm represents high-dimensional data in two or three-dimensional space, ensuring that the mapped data points and their neighboring data maintain analogous spatial relationships to those observed in the original space. The t-SNE algorithm provides a straightforward visualization of the classification problem. If the data are effectively separated in the low-dimensional space, the model can readily distinguish between them. Conversely, if the data are clustered together in the low-dimensional space, the model exhibits reduced classification accuracy. Lee et al. examine the impact of model classification accuracy on t-SNE [123]. The network they used for high-entropy alloy phase classification has a total of five hidden layers. In the first three hidden layers, the differences between the data are not well discriminated, the points in t-SNE are still clustered together, and the classification accuracy of the model is not high. After going through all five hidden layers, the classification accuracy of the model reaches 93.17%, and the points in the t-SNE are also separated (Figure 5) [123].

**Figure 6:**
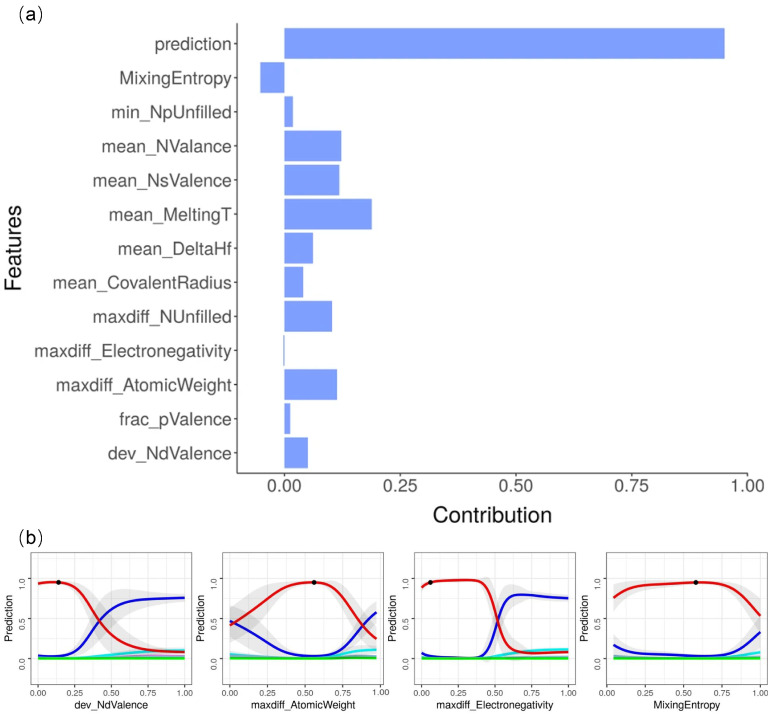
Analysis of model interpretation in Ref. [124]: (**a**) the contributions of descriptors for BCC phase in the NbTaTiV system; (**b**) phase predictions when there is only one descriptor change. Line colors denote phase information: blue, mixed phases; violet, AM; cyan, FCC; orange, BCC + FCC; light blue, HCP; red, BCC; green, IM.

Lee et al. develop an interpretable machine learning model for phase classification of HEAs based on the contribution of descriptors [124]. In their breakdown (BD) approach, the model predictions for a single observation are decomposed into contributions attributable to different input variables. Furthermore, the contributions of descriptors to different phases are separated to obtain a local contribution map. The mixing entropy of NbTaTiV has a negative contribution (see Figure 6a). The mean_MeltingT, mean_NValence, and mean_NsValence variables are of particular significance, as they exert the greatest influence on the formation of components within the BCC phase. The probability of each phase predicted by the machine learning model in the NbTaTiV system when there is only one descriptor change can help to study the dependence of predictions on specific descriptors, as shown in Figure 6b.

In addition to interpretability, the analysis of the results should also encompass the capacity to generalize and the overfitting issues, particularly in the context of small datasets. Five-fold cross-validation is a commonly used method for model validation [125]. A good machine learning algorithm should ensure that there is little difference in the accuracy of the five models. This is to prevent particular data splits from causing the models to show falsely high performance with low generalization. Victor et al. propose a method of cross-validation of classification and regression models to determine SISSO descriptors to avoid the overfitting problem of traditional SISSO algorithms in HEAs, which successfully improves the performance of the models on the test set [126].

Experimental validation is the most effective test for the performance of machine learning. Li et al. validate 18 HAEs in experimental articles [127]. The phases of the 13 HEAs are consistent with the machine learning results. The experimental results of Chen et al. are consistent with machine learning predictions of eutectic alloys [128]. All experimentally synthesized HEAs are intended eutectic alloys.

## 3. Special Machine Learning Algorithms

Some machine learning algorithms differ in function or process from general machine learning algorithms. For instance, there are generative models, which serve the complemented purpose to predictive models. Other special algorithms, such as data augmentation algorithms and transfer learning, can enhance the predictive performance of models. We will describe each of them in this section.

### 3.1. Generative Models

The application of predictive machine learning models necessitates the input of elemental ratios or structural information in order to obtain material properties. As the number of possible combinations increases exponentially with the range of elemental species, the process of global screening of material properties can become a large and complex task. To overcome these problems generative machine learning models are proposed [129]. In contrast to predictive models, generative models receive the desired material properties and provide combinations directly from the potential material space. Such models are capable of identifying and extracting hidden patterns from complex databases, subsequently utilizing them to generate novel combinations of structures that satisfy specific attribute requirements, negating the necessity for additional human input [130,131]. The most commonly utilized generative models include Generative Adversarial Network (GAN) [132], Variational Autoencoder (VAE) [133], flow-based model [134], diffusion model [135], and so on.

**Figure 7:**
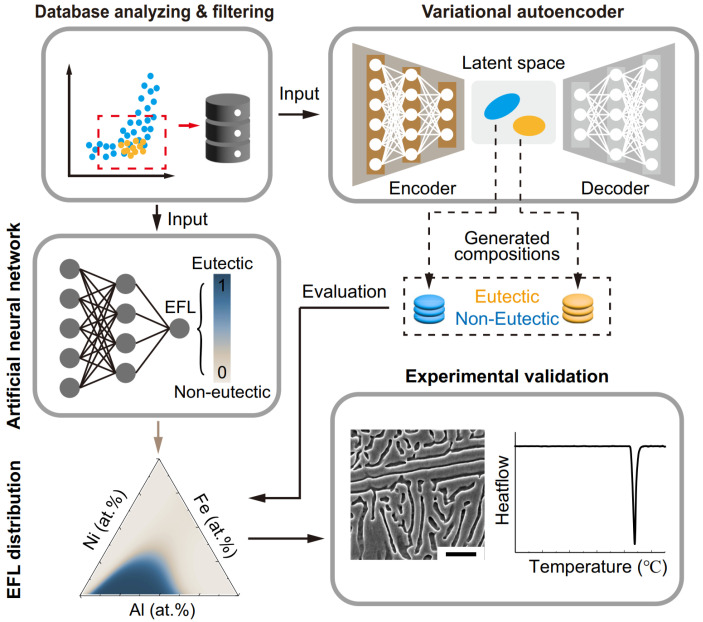
Algorithm process and generated results in Ref. [128]: VAE and ANN work together to ensure the effectiveness of generating eutectic alloys; possible eutectic alloy components are generated by machine learning and experimentally verified.

Generative models have been successfully used in HEAs. Li et al. used GAN to generate single-phase HEAs and obtained 188 potential materials with densities of less than 8 $g/{\mathrm{cm}}^{3}$ and prices of less than $USD 12/g [127]. A VEA model for generating complex eutectic alloys is developed by Chen et al. [128] The process of their algorithm is illustrated in Figure 7. The encoder network maps the input components and descriptors into a two-dimensional potential space, where the eutectic and non-eutectic components are classified into two distinct groups. Subsequently, the decoder network generates combinations based on the corresponding groups in the potential space. The quality of the generated data is also evaluated in conjunction with an ANN model for predicting eutectic alloys. The potential eutectic compositions are generated ternary alloy combinations, which are then observed under a scanning electron microscope. The experimental results demonstrate that VAE was effective in generating potential eutectic alloys.

### 3.2. Data Augmentation

Notwithstanding the rapid evolution of methodologies and computational resources, the implementation of DFT computations remains a costly and inefficient process. This has resulted in datasets typically being limited to less than 10,000 for the development of material machine learning models, with datasets larger than this size being computationally prohibitively expensive. The advantage of machine learning models in terms of computational cost is no longer a factor. Furthermore, the potential material space for multi-component materials like HEAs is vast [58], and the limit of the dataset has become a significant challenge that impedes the accuracy of the models.

The method of increasing the number of samples in a dataset by processing the original data in the dataset and adding data containing new information is known as data augmentation. Ye et al. add Gaussian noise to the original data and fed these noisy data into the model to train it as data augmentation [136]. This approach achieved good results in predicting the hardness of HEAs. In addition, Ye et al. show the effect of the amount of augmented data and the amount of noise on the model performance, as given in Table 2. The data-augmented model has good performance on the validation set, which the model has never seen before, suggesting that data augmentation has better performance away from the training set and improves the generalization of the model. The middle noise-enhanced model performs best, while too much enhanced data degrades model performance.

Generative models such as GAN can also be used for data augmentation. Chen et al. develop a data-augmented model for the classification of phases in HEAs using data generated by the GAN model [137]. The classification accuracy of the data-augmented model reaches 96.08%.

### 3.3. Transfer Learning

Transfer learning represents a valuable machine learning technique that facilitates the sharing of knowledge between domain models that are related in some way [138]. Transfer learning can also address the problem of insufficient datasets. Transfer learning typically pre-trains the network on a large, easily accessible dataset, after which it keeps some of the network structure and parameters unchanged and trains the model on a small dataset. Wang et al. reduce the mean absolute error for predicting magnetic moments of HEAs from 0.197 $\mu_{B}/\mathrm{atom}$ to 0.091 $\mu_{B}/\mathrm{atom}$ by pre-training the model on the binary and ternary alloys [97].

**Figure 8:**
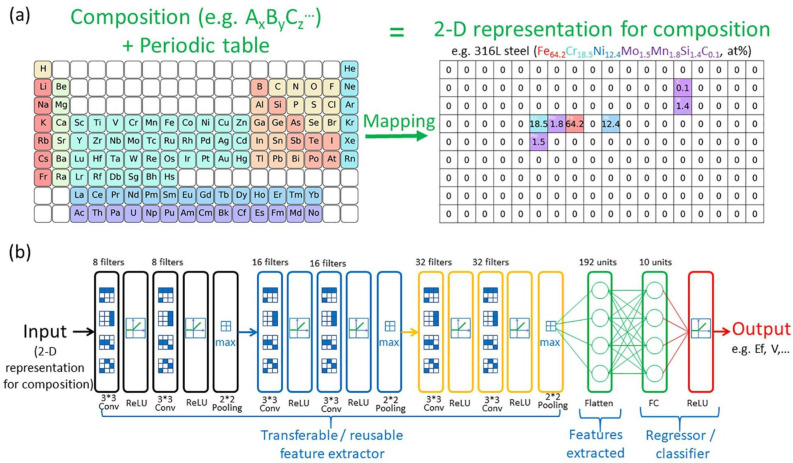
Algorithm process in Ref. [75]: (**a**) Mapping the chemical formula of materials to a two-dimensional representation that employs the periodic table structure. (**b**) Network structures of the model, containing the transferable feature extractor and separately trained regressor or classifier.

Feng et al. propose a transfer learning algorithm based on CNN [75]. The process of their transfer algorithm is shown in Figure 8. They regard the convolutional layer of CNN as a feature extractor, capable of extracting generic material feature representations from diverse materials suitable for machine learning. These elemental features are transferable. They train the feature extractor on 228,676 compounds, followed by a phase classification model trained on two HEA datasets. The classification accuracies on the two datasets are 0.93 and 0.939, respectively. It shows that the feature extractor captures the generic elemental features well and can help machine learning models on small datasets to achieve better performance [75].

## 4. Challenges and Future Directions

Today, machine learning still faces many challenges in the field of high-entropy alloys. The past applications of machine learning in the field of high-entropy alloys predominantly concentrate on the classification of phases and the prediction of mechanical properties. Using machine learning to capture more underlying physical properties such as formation energy and magnetic moments is one of the future directions. Unlike the mechanical properties, these underlying physical properties vary dramatically with elemental components and are strongly correlated with the structure of alloys. The inclusion of more underlying physical information such as translation and rotation symmetries in crystal structures in machine learning descriptors is a key ingredient for the successful prediction of properties such as formation energies. However, the introduction of structure means that more calculations are required in preparing the training set. Balancing the consumption of developing algorithms with the accuracy of the algorithms is also a problem that needs to be focused on. At the present, the idea of using machine learning to capture the underlying physical information is currently being successfully applied to assist molecular dynamics simulations. The accuracy of predicting interatomic potentials is improved by constructing machine learning potentials that satisfy translational invariance and rotational covariance or by using graph neural networks directly to satisfy translational invariance and rotational covariance [42].

Another challenge is obtaining high-quality datasets. Both experiments and simulations to obtain data on high-entropy alloys are time consuming. Experimental or simulation data from different researchers often have the problem of being obtained under different conditions. It is difficult to calibrate these data. As a result, datasets for high-entropy alloys are often small. However, due to the large component space of high-entropy alloys, small datasets often cause more problems than in other materials. Although we show in Section 3 that generative models and data augmentation are used to improve prediction accuracy with small datasets, these methods do not fully address the problems posed by small datasets. In the future development of machine learning models, it is important to construct a uniform and accurate dataset. In addition, the mechanical properties of high-entropy alloys are affected by defects, dislocations, and so on. More materials containing defects and dislocations should be included in the machine learning datasets.

The third problem is the reliability of the generative model. Generative models such as GANs often rely on the similarity of the original data to generate new data. Currently, the generative model is mainly applied to generate single-phase high-entropy alloys. This is due to the fact that high-entropy alloys of the same phase tend to have similar structures. However, the relationship between structure and properties of materials often does not follow similarity. Identical properties may correspond to completely different structures, and similar structures may have large differences in properties. Therefore, the properties obtained using generative models are usually used for data augmentation to improve prediction accuracy [137]. In the meantime, the intricate and fluctuating nature of high-entropy alloys renders it challenging for directly generative models to meet the requisite mechanical properties or magnetic moments. In order to satisfy the need of inverse material design from properties to structures, the use of generative models to obtain structures that satisfy performance requirements is a major future challenge.

## 5. Conclusions

The synergy between machine learning and materials science has fostered the emergence of a new, exciting, and exponentially growing field. High-entropy alloys are particularly interesting candidates for demonstrating the power of machine learning due to their superior mechanical properties, vast compositional space, and complex chemical interactions. Models applicable to high-entropy alloys continue to be proposed in various parts of the machine learning process. Some of the works take a data or computational algorithmic perspective to improve model performance. Others focus on the unique properties of high-entropy alloys, such as exploring unique representations of the short-range and long-range order of high-entropy alloys and seeking more physically correct representations. These efforts have greatly improved the effectiveness of machine learning in high-entropy alloys. The unique large-scale screening capability of machine learning is also providing new impetus for the development of high-entropy alloys. In addition, interpretable machine learning models in high-entropy alloys have also progressed, bringing unprecedented directions. Generative models are capable of providing potential element proportions and structures, thereby greatly reducing the difficulty of predictive model search in high-dimensional spaces. The incorporation of data augmentation and transfer learning offers a novel avenue for enhancing the performance of prediction in high-entropy alloys with limited data. Overall, machine learning is well developed in the field of high-entropy alloys, but there are many exciting and challenging problems to be solved.

## Figures and Tables

**Table 1:** RMSE for predicting hardness of HEAs on the test set of different models.

**Model**	SVM	KNN	ANN
RMSE *	82	69	65

* The data of RMSE is from Ref. [90].

**Table 2:** RMSE for predicting hardness of HEAs on the test set and validation set with Gaussian noise as data augmentation: The test set is the test data split from the original dataset. The validation data are data not in the original set that the model has never seen before.

Augmented Data	RMSE in Test Set *	RMSE in Validation Set *
Row Data	58.1	44.4
2 × Row Data	42.8	40.5
Low noise enhanced	42.8	40.1
Middle noise enhanced	43.2	39.6
High noise enhanced	43.7	40.0
3 × Row Data	39.0	41.5
Low + middle noise enhanced	39.8	41.0
Low + high noise enhanced	40.0	40.7
Middle + high noise enhanced	40.9	40.5
4 × Row Data	30.2	43.1
Low + middle + high noise enhanced	31.1	41.5

* The data of RMSE are from Ref. [136].

## Abbreviations

The following abbreviations are used in this manuscript:

HEA	High-entropy alloys
RHEA	Refractory high-entropy alloys
SRO	Short-range order
LRO	Long-range order
DFT	Density functional theory
MD	Molecular dynamics
CALPHAD	Phase diagram calculation
AI	Artificial intelligence
EPI	Effective pair interaction
SISSO	Sure independence screening and sparsifying operator
AM	Amorphous
IM	Intermetallic
SS	Solid solution
BCC	Body-centered-cubic
FCC	Face-centered-cubic
VASE	Voronoi analysis and Shannon entropy
$R^{2}$	Coefficient of determination
SVM	Support vector machine
CART	Classification and regression tree
KNN	k-nearest neighbor
ANN	Artificial neural network
CNN	Convolutional neural network
RNN	Recurrent neural network
RMSE	Root mean square error
GNN	Graph neural network
ECNet	Elemental convolution graph neural network
SHAP	SHapley Additive exPlanations
BD	breakdown
t-SNE	t-distributed stochastic neighbor embedding
GAN	Generative adversarial network
VAE	Variational autoencoder
